# Synthesized Nano-Titanium Dioxide (Nano-TiO_2_) via Ammonium Fluorotitanate ((NH_4_)_2_TiF_6_) Precipitation with Ammonia Solution

**DOI:** 10.3390/nano15120930

**Published:** 2025-06-15

**Authors:** Yufeng Guo, Cong Zhou, Shuai Wang, Feng Chen, Yanqin Xie, Jinlai Zhang, Lingzhi Yang

**Affiliations:** School of Minerals Processing and Bioengineering, Central South University, Changsha 410083, China; yfguo@csu.edu.cn (Y.G.);

**Keywords:** titanium dioxide, nanoparticle, preparation, photocatalysis

## Abstract

This study focuses on the chemical synthesis of nano-titanium dioxide (nano-TiO_2_) via ammonium fluorotitanate ((NH_4_)_2_TiF_6_) precipitation with ammonia solution, aiming to elucidate the effects of experimental parameters—including reaction temperature, duration, molar ratio of (NH_4_)_2_TiF_6_ to ammonia, and (NH_4_)_2_TiF_6_ concentration—on the particle size of synthesized nanoparticles, as well as the correlation between particle size and photocatalytic performance. The synthesized nanoparticles predominantly exhibited spindle-shaped morphology. Direct TEM imaging revealed the crystallization and growth mechanisms during synthesis: higher molar ratios, combined with lower temperatures and shorter durations, facilitated the formation of ultrafine particles, whereas lower molar ratios, with elevated temperatures and prolonged reaction times, yielded larger particles. Notably, nanorod structures emerged under low-temperature conditions with F^−^ ion adsorption. To investigate the relationship between particle size and photocatalytic performance, a Taguchi method-inspired experimental design was employed to evaluate the positive or negative impacts of particle size on photocatalytic activity. An experimental matrix was constructed using coded values for each factor, and regression coefficients were calculated to quantify input-output correlations. Results demonstrate that titanium dioxide catalysts with a particle size range of 50–75 nm exhibit optimal photocatalytic efficiency.

## 1. Introduction

Industrial dye pollutants (e.g., Rhodamine B, Methylene Blue, Methyl Orange) have emerged as a significant global challenge in water environment remediation due to their high chromaticity, acute toxicity, and recalcitrant degradation [1,2,3,4]. Statistical data indicate that China annually discharges over 160 million cubic meters of textile dyeing wastewater, with approximately 10–15% of organic dyes directly entering aquatic systems, inducing ecotoxicity and posing threats to human health [5]. Conventional biological treatment methods exhibit limited efficacy in degrading dye molecules containing polycyclic aromatic structures. In contrast, photocatalysis technology, which drives redox reactions via photoinduced charge carriers in semiconductor materials, can mineralize pollutants into harmless substances such as CO_2_ and H_2_O, thereby establishing itself as a cutting-edge solution to this critical issue. Beyond dye pollutants, photocatalysis has been demonstrated as an effective technology for treating emerging contaminants, including pharmaceuticals (Diclofenac [6,7], Sulfadiazine [8,9]), phenolic compounds (Bisphenol A [10,11]), and heavy metal ions [12].

Titanium dioxide (TiO_2_), as a classical photocatalyst, has demonstrated significant potential in pollutant degradation. Conventional precursors for the synthesis of nano-TiO_2_ typically include titanium alkoxides [13,14], titanium tetrachloride [15], and metatitanic acid [16]. Zhu et al. synthesized TiO_2_ via a non-hydrolytic sol-gel process using titanium sulfate and benzyl alcohol at low temperatures, evaluating the photocatalytic degradation of phenol [13]. Hoa et al. prepared vertically aligned carbon-Fe: TiO_2_ nanobelts via vapor-phase decomposition using a titanium alkoxide precursor (Diisopropoxybis(2,4-pentanedionate)titanium), reporting applications in photocatalysis, solar cells, and lithium-ion batteries [14]. Cai et al. produced TiO_2_ nanoparticles by gas-phase high-temperature hydrolysis in a H_2_/O_2_ flame reactor using TiCl_4_, achieving uniform particle size (16–34 nm) and tunable rutile content (9–81%) through parameter optimization [15]. Bao et al. synthesized rutile TiO_2_ via thermal decomposition of metatitanic acid, optimizing microwave calcination using response surface methodology [16]. Compared with these conventional titanium sources, (NH_4_)_2_TiF_6_ as a titanium source exhibits multiple advantages for nanomaterial fabrication: its low-temperature decomposition behavior, fluoride-mediated morphological control (F^−^ ions enable the formation of mesoporous structures and guide single-crystalline nanowire or nanosheet architectures), high-purity product characteristics (absence of Cl^−^/${SO}_{4}^{2-}$ residues), and environmental compatibility through closed-loop exhaust treatment. Furthermore, this compound demonstrates exceptional solubility in both aqueous and acidic media, facilitating rapid formation of homogeneous reaction solutions. These properties effectively minimize local concentration gradients, suppress particle agglomeration, and enhance product dispersibility without requiring complex dispersion protocols. Such combined merits make it particularly suitable for large-scale continuous production, achieving an optimal balance between energy efficiency and precise structural control.

The liquid-phase precipitation method exhibits remarkable advantages in fabricating nano-titanium dioxide, making it particularly suitable for low-cost, large-scale industrial applications [17]. Compared to physical approaches involving uneven particle sizes from mechanical grinding and the high expenses of physical vapor deposition (PVD) [18], chemical vapor deposition’s high-temperature corrosion and energy intensity [19], as well as other liquid-phase methods such as the instability of microemulsion systems [20], multistep complexities in sol-gel processes [21], and high-pressure equipment requirements for hydrothermal synthesis [22], the hydrolysis-precipitation method simplifies processing through ambient-condition liquid-phase reactions. This approach eliminates the need for expensive equipment and elevated temperatures while offering lower raw material costs and enhanced scalability. Moreover, its operational simplicity (precipitation → washing → drying) surpasses techniques requiring precise control, such as electrospinning [23] or specific interdisciplinary hybrid methods [24,25,26]. Although precipitation faces limitations including broad particle size distribution and strict pH control requirements, its economic viability and practicality remain particularly prominent in applications tolerant of moderate size variations [27].

The precipitation method is often combined with the pyrolysis method to prepare titanium dioxide nanomaterials, that is, further heat treatment after precipitation to obtain titanium dioxide. Sanchez-Martinez et al. used Ti(iPr)_4_ and NH_4_OH for co-precipitation, followed by calcination in air at 200, 300, 400, and 500 °C for 2 h to obtain nano-titanium dioxide with similar morphology but different particle sizes. They found that the sample treated at 500 °C had the largest particle size and the best photocatalytic performance [28]. Yeh et al. used TiCl_4_ and NH_4_OH to precipitate titanium dioxide, which resulted in a residual NH_4_Cl that could only be removed after calcination [29]. Liu et al. used Ti(SO_4_)_2_ and urea for precipitation, then calcined the material at 550 °C for 4 h to obtain titanium dioxide, and studied its antibacterial properties against *E. coli* under light irradiation [30]. Ammonium fluorotitanate does not require a heat treatment process and can be directly converted into nano-titanium dioxide through liquid-phase precipitation, thereby significantly reducing energy consumption and improving production efficiency.

This study used ammonia as the precipitant to directly precipitate nano-sized titanium dioxide from an (NH_4_)_2_TiF_6_ solution. Under alkaline conditions, the [TiF_6_]^2−^ ions in the aqueous (NH_4_)_2_TiF_6_ solution undergo stepwise hydrolysis and hydroxylation reactions, ultimately forming amorphous or crystalline TiO_2_ precursors or precipitates, as described in reaction (1). Theoretical stoichiometric analysis indicates that a 1:4 molar ratio of (NH_4_)_2_TiF_6_ to hydroxide ions satisfies the stoichiometric requirements for complete reaction. By controlling precipitation parameters such as pH, temperature, and the hydrolysis/dissociation kinetics of the fluoro-titanium complex, the precise regulation of TiO_2_ nucleation and growth kinetics can be achieved, resulting in highly dispersed nanostructures. Notably, the small size of F^−^ ions allows them to easily dope into the TiO_2_ lattice or substitute for some lattice oxygen, forming defects such as titanium-oxygen vacancies. These defects (e.g., oxygen vacancies) can act as traps for photogenerated electrons and holes or alter the local electronic structure, thereby enhancing the separation efficiency of photogenerated charge carriers (electron-hole pairs). Additionally, fluoride ions (F^−^) adsorbed on specific crystal facets during precipitation can inhibit Ostwald ripening and induce the formation of surface defects, further improving the separation efficiency of photogenerated charge carriers.

(1) $$[TiF_{6}{]}^{2-}+4OH^{-}\to TiO_{2}↓+6F^{-}+2H_{2}O$$

The pH value has a significant influence on the morphology and crystallization behavior of titanium species. The (NH_4_)_2_TiF_6_ solution itself is weakly acidic. When the pH is below 3, the [TiF_6_]^2−^ complex ion remains stable under the strong coordination of F^−^ ligands, without significant hydrolysis. Ammonia water, as an inorganic weak base, gradually releases OH^−^ ions in the solution. When the pH exceeds 3, the OH^−^ concentration may become sufficient to compete with F^−^ ligands, triggering the gradual hydrolysis of [TiF_6_]^2−^. This process involves the partial substitution of F^−^ ligands by OH^−^, forming mixed-ligand fluoro-hydroxy complexes (Reaction (2)). As the pH increases, the OH^−^ concentration rises significantly, leading to complete ligand substitution, where OH^−^ gradually replaces F^−^. Consequently, the titanium species transform into a hydroxyl-dominated coordination structure (Reaction (3)) [31]. The hexahydroxotitanate ion [Ti(OH)_6_]^2−^ is highly unstable in solution. Titanium(IV) has a high charge density, which promotes the dehydration condensation (olation) of coordinated water or hydroxyl groups, forming oligomers or polymers containing oxo bridges (O-Ti-O). It is typically only a transient intermediate or an idealized species that does not exist. [TiO(OH)_5_]^−^ is also not a typical final product or representative of polymerization; Reactions (2) and (3) merely illustrate the principles of the reaction process, with the complete reaction process described by Reactions (4) and (5). By controlling the reaction conditions, TiO_2_ crystals can be directly precipitated; however, their crystallinity generally does not reach full perfection.

(2) $$\left[TiF_{6}{]}^{2-}+nOH^{-}⇌[Ti(OH{)}_{n}F_{6-n}{]}^{2-}+{nF}^{-}(n=1–6\right)$$

(3) $$[Ti{(OH{)}_{n}F}_{6-n}{]}^{2-}+(6-n)OH^{-}\to [Ti(OH{)}_{6}{]}^{2-}polymerize\to [TiO(O{H)}_{5}{]}^{-}$$

(4) $$[Ti{(OH{)}_{n}F}_{6-n}{]}^{2-}+(6-n)OH^{-}\to Ti(OH{)}_{4}\left(aq\right)+\left(6-n\right)F^{-}+(2-n)H_{2}O$$

(5) $$mTi{\left(OH\right)}_{4}\to [TiO_{4}\cdot (H_{2}O{)}_{x}{]}_{m}\left(Amorphous\;gel\;or\;precipitate\right)\;or\to TiO_{2}(Crystallization)+2mH_{2}O$$

Under conditions of sufficient OH^−^ supply, the accelerated hydrolysis of titanium species theoretically drives the condensation reaction along the lowest energy pathway, preferentially forming stable anatase nuclei. The pH value directly governs the nucleation-growth balance and crystallization process of nano-TiO_2_ by regulating the coordination chemistry of titanium species. A uniform pH environment is crucial for obtaining monodisperse, highly crystalline anatase; the key to experimental optimization lies in controlling mixing efficiency and reaction kinetics. Throughout the process, F^−^ stabilizes intermediates through strong Ti-F bonds (bond energy ~560 kJ/mol, significantly higher than that of Ti-OH bonds at ~250 kJ/mol), thereby inhibiting the ordered arrangement of nuclei. Increasing the pH promotes the cleavage of Ti-F bonds, shifting the equilibrium toward product formation (Reaction (6)).

(6) $$[TiF_{6}{]}^{2-}+2H_{2}O⇌TiO_{2}+6F^{-}+4H^{+}$$

There are relatively few studies on the preparation of nano-titanium dioxide using ammonium fluorotitanate as a raw material via the precipitation method, with most of them employing boric acid as the precipitant. Boric acid solutions are weakly acidic and differ from the principle of reaction with bases. Boric acid removes F^−^ through complexation (the B-F bond has high bond energy, approximately 757 kJ/mol, and short bond length, resulting in extremely high stability), driving the hydrolysis of [TiF6]^2−^ with H_2_O to form TiO_2_, rather than following the OH^−^ substitution pathway in alkaline solutions. Lei et al. directly precipitated TiO_2_ films using (NH_4_)_2_TiF_6_ and H_3_BO_3_ under different parameters and found that concentrations of 0.03 M and 0.09 M, respectively, reacted at 80 °C for 3 h in a solution with pH 2.9 produced TiO_2_ films that provided the most effective photogenerated cathodic protection for 304 stainless steel [32]. Wang et al. prepared Ti_3_C_2_/TiO_2_ composite materials via liquid-phase precipitation using (NH_4_)_2_TiF_6_, Ti_3_AlC_2_, and H_3_BO_3_. Through photodegradation experiments of methylene blue under UV irradiation, they demonstrated that the photocatalytic performance of the Ti_3_C_2_/TiO_2_ composite was superior to that of commercial anatase TiO_2_ nanoparticles [33]. Chen et al. utilized (NH_4_)_2_TiF_6_ and boric acid (H_3_BO_3_) solution to deposit TiO_2_ films on polystyrene (PS) microsphere templates via liquid-phase deposition (LPD). After high-temperature sintering to remove the PS microspheres, TiO_2_ hollow layers were prepared, and their excellent performance in dye-sensitized solar cells (DSSCs) was verified [34]. Examples of research using alkaline precipitants are scarce. Xu et al. designed a porous, dispersed double-T-type micromixer and employed liquid-phase deposition (LPD) with (NH4)2TiF6 and CO(NH_2_)_2_ as reactants to directly precipitate ordered TiO_2_ films at low temperatures within the reactor [35]. This demonstrated the advantages of micromixing technology, providing a practical approach for film preparation in liquid-liquid reaction systems with rapid precipitation processes.

In this study, ammonia was used as the precipitant to directly precipitate well-crystallized nano-titanium dioxide particles at extremely low reaction concentrations. The photocatalytic degradation performance of rhodamine B was evaluated to assess the influence of synthesis parameters during precipitation on particle morphology and size, which was the core focus. Transmission electron microscopy (TEM) imaging directly revealed the morphology and size distribution of the nanoparticles, further elucidating the impact of particle size on the photocatalytic degradation efficiency of rhodamine B. The experimental method is novel and can provide fundamental data for research on the precipitation of nano-titanium dioxide under alkaline conditions, offering new insights for the development of highly efficient and stable photocatalysts.

## 2. Experimental Section

### 2.1. Materials and Characterization

Ammonium hexafluorotitanate ((NH_4_)_2_TiF_6_, 98%), analytical-grade ammonia solution (25–28%), Rhodamine B (≥99.0%), and commercial TiO_2_ nanoparticles (P25) were obtained from Shanghai Macklin Biochemical Technology Co., Ltd. in China (Shanghai, China). Ultrapure water (resistivity 18.2 MΩ·cm) was used throughout the experiments.

All samples were characterized using a field-emission transmission electron microscope (FE-TEM; JEM-F200, JEOL Ltd., Akishima, Tokyo, Japan) operated at an acceleration voltage of 200 kV to observe their microstructures. Concurrently, X-ray diffraction (XRD; D8 Advance, Bruker Corporation, Billerica, DE, USA) analysis was performed over a 2θ range of 5–80° with a step size of 0.02°. The acquired XRD patterns were analyzed using MDI Jade 9.0 software, using the PDF-4+ 2009 database for crystal structure determination and phase composition identification. The crystallinity of the samples was jointly confirmed by XRD diffraction patterns and high-resolution transmission electron microscopy (HRTEM) images.

Since the preparation procedures for all samples were identical to those of the raw materials but with different parameters, we selectively performed X-ray photoelectron spectroscopy (XPS) analysis on three samples exhibiting significant differences in photocatalytic performance to gain insight into the elemental composition and chemical states of all samples. The XPS measurements were conducted on a Shimadzu Kratos AXIS Supra+ spectrometer, using a monochromatic Al Kα excitation source (1486.6 eV), with a base vacuum of better than 5 × 10^−9^ mbar. All binding energies were calibrated against the C 1s peak (284.8 eV) of surface-contaminated carbon. Data analysis and spectral fitting were performed using Avantage 6.8.0 software.

Absorbance measurements in the photocatalytic experiments were conducted using a UV-Vis spectrophotometer (UV-2600 Shimadzu Corporation, Nakagyo-ku, Kyoto, Japan).

### 2.2. Preparation of Nano-TiO_2_

**Figure 1:**
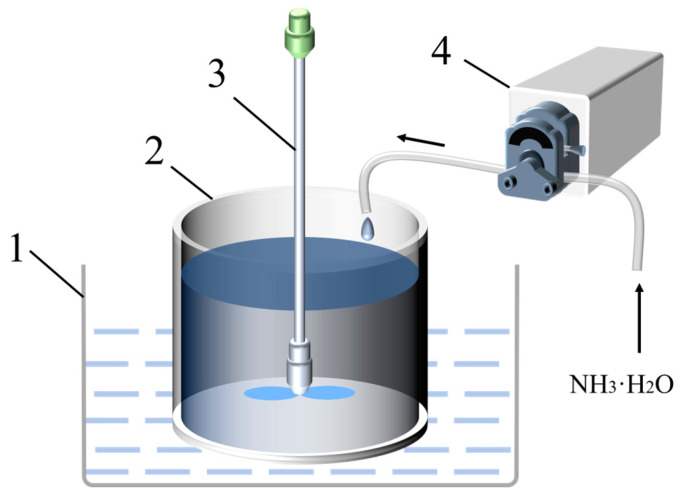
Experimental setup for nano-TiO_2_ precipitation synthesis (1: constant-temperature oil bath; 2: reaction beaker; 3: mechanical stirrer; 4: peristaltic pump).

A 500 mL aliquot of (NH_4_)_2_TiF_6_ solution with a predetermined concentration was transferred into a beaker and thermally regulated to the target temperature using an oil bath. Subsequently, 500 mL of ammonia solution with controlled concentration was titrated into the reaction system at a constant rate via a peristaltic pump, while maintaining mechanical agitation at 400 rpm. The ammonia concentration was adjusted according to the preset molar ratio of (NH_4_)_2_TiF_6_ to precipitant, with reaction duration (i.e., total titration time) governed by precise pump speed modulation. Upon completion of the reaction, the suspension was immediately subjected to solid-liquid separation via high-speed centrifugation (10,000 rpm, 10 min). The precipitate underwent five cycles of centrifugal washing with deionized water (under identical conditions) until the supernatant conductivity reached below 5 μS·cm^−1^ (25 °C), ensuring complete removal of residual electrolytes. The washed sample was vacuum-dried at 60 °C for 12 h. Aggregates formed by capillary forces during drying were pulverized using an agate mortar, resulting in a homogeneous powder suitable for subsequent characterization and evaluation of photocatalytic performance. The experimental setup is shown in Figure 1. A total of 20 samples, labeled S1 through S20, were synthesized in this study. The synthesis parameters and sample numbers are detailed in Supporting Material Table S1.

### 2.3. Catalytic Experiments

**Figure 2:**
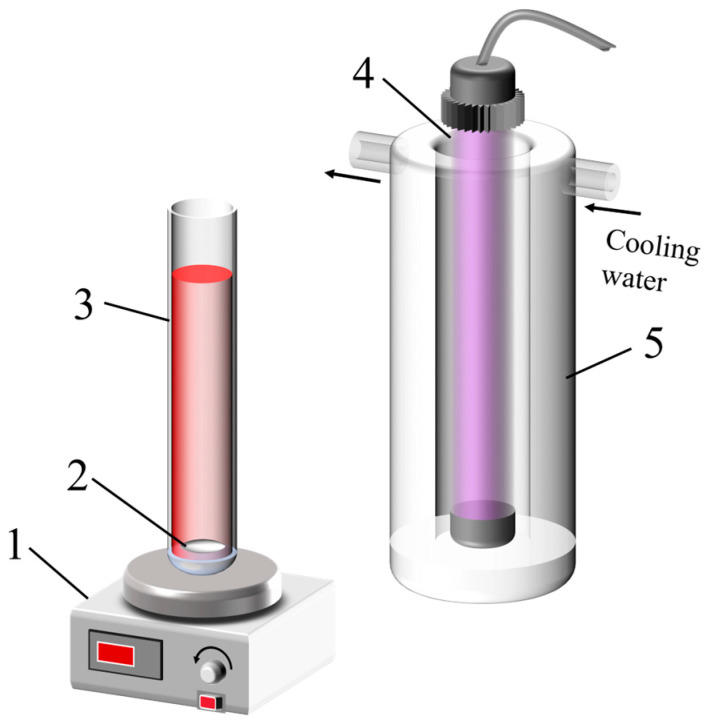
Photocatalytic reaction apparatus (1: magnetic stirrer; 2: magnetic stirring bar; 3: quartz reactor tube; 4: light source; 5: quartz cold trap).

The photocatalytic reaction was conducted using the apparatus shown in Figure 2. A quartz tube (15 mm diameter) served as the reaction vessel, positioned 5 cm from the light source and cold trap. Before the experiment, the initial absorbance of a 20 mg L^−1^ Rhodamine B solution (denoted as A0) was measured at its maximum absorbance wavelength of 554 nm, see (Figure S2 in the Supplementary Information). Subsequently, 25 mL of the Rhodamine B solution and 0.01 g of nano-TiO_2_ catalyst (equivalent to 0.4 g L^−1^ catalyst loading) were sequentially introduced into the quartz tube. The mixture underwent 10 min of ultrasonication in complete darkness to ensure uniform dispersion of the catalyst. After dark adaptation, the quartz tube was repositioned in the experimental setup, and photocatalytic degradation was initiated under irradiation from a low-pressure mercury lamp (365 nm wavelength, 30 W power) for 1 h. Continuous mixing at 120 rpm was maintained via a magnetic stirrer, with the entire reaction system enclosed in a light-tight chamber. Following the reaction, the suspension was centrifuged at 10,000 rpm for 10 min to achieve phase separation. The absorbance of the supernatant was also measured at a wavelength of 554 nm (denoted as A). The photocatalytic degradation efficiency (η) was calculated using the formula: η = [(C0 − C)/C0] × 100% = [(A0 − A)/A0] × 100% (where C and C0 represent the concentration after degradation and the initial concentration, respectively).

Before conducting formal experiments, the photocatalytic performance of commercially available P25 nano-TiO_2_ (hereafter referred to as P25) was validated. After a 10-min ultrasonic dispersion, photocatalytic degradation experiments were performed for 0.5 h, 1 h, 1.5 h, and 2 h, yielding degradation efficiencies of 28.03%, 42.92%, 56.57%, and 89.57%, respectively, see (Table S4 in the Supporting Information). Considering degradation kinetics and operational efficiency, the formal experimental protocol was established as follows: Rhodamine B solution was ultrasonically dispersed with a catalyst for 10 min, followed by a 1-h photocatalytic period. This design provides an adequate experimental window for both suboptimal and exceptional photocatalytic scenarios while maintaining optimal resource utilization.

### 2.4. Experimental Design Method

The primary objective of this study is to investigate the impact of process parameters on the size and photocatalytic performance of nano-titanium dioxide particles. To analyze the effect of process parameters on particle size, systematic transmission electron microscopy (TEM) direct imaging and analysis of crystallization behavior were conducted. In investigating the impact of particle size on photocatalytic performance, a Taguchi method-inspired experimental design was employed [36,37]. This method systematically evaluates the impact of key parameters (such as (NH_4_)_2_TiF_6_ concentration and reaction temperature) on single or multiple response factors by analyzing a series of well-defined synthetic process data. Detailed information about this method is presented in Section S1.1 of the Supporting Information.

## 3. Results and Discussion

### 3.1. Characterization

More than half of the samples were characterized by XRD to verify the crystal phase, crystallinity, and purity of the synthesized catalysts. The results confirmed that the diffraction peaks of all tested samples corresponded to the anatase phase (PDF-4+ 2009 database, Card No. 01-075-2553), with no significant impurity peaks observed. The HRTEM images of samples S2 and S17 display clear lattice fringes (Figures S4 and S5), indicating the crystallinity of the samples to some extent. A varying degree of amorphous hump was detected at 2θ = 10–20°, indicating the presence of amorphous materials [TiO_4_·(H_2_O)_x_]_m_. XPS analysis was performed on a representative sample S2, revealing the presence of fluorine and nitrogen residues in the material (Figure S6).

Transmission electron microscopy (TEM) characterization results of all samples are presented in Figure S3. Scale bars (100 nm) in the images reveal the nanoparticle morphology and size distribution. All titanium-based particles exhibit spindle-shaped morphology, while Sample S9 additionally displays rod-like structures. The spindle morphology arises from the critical role of F^−^ ions (generated via (NH_4_)_2_TiF_6_ decomposition) as structure-directing agents. F^−^ is released from the stable complex (Equation (4)) and preferentially adsorbs on the high-energy {001} crystal plane of anatase TiO_2_. This adsorption suppresses growth rates along the {001} direction, whereas the F^−^-uncovered {101} facets exhibit accelerated growth, leading to anisotropic crystal elongation and spindle formation.

#### 3.1.1. The Effect of the Molar Ratio of (NH_4_)_2_TiF_6_ to Ammonia

**Figure 3:**
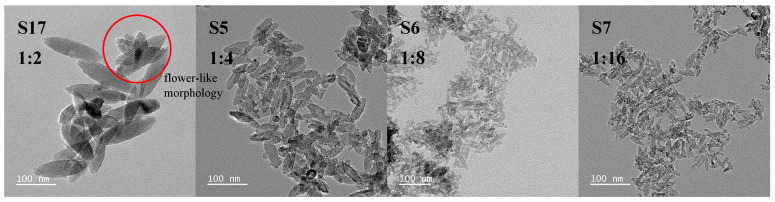
TEM images of spindle-shaped TiO_2_ nanoparticles synthesized under different molar ratios of (NH_4_)_2_TiF_6_ to ammonia solution (sample numbers S17, S5, S6, S7). Constant parameters maintained: (NH_4_)_2_TiF_6_ concentration = 0.01 mol·L^−1,^ reaction temperature = 90 °C, reaction time = 1 h. Note the multi-layered, flower-like morphology aggregated from nanosheets in sample S17. Scale bar: 100 nm.

**Figure 4:**
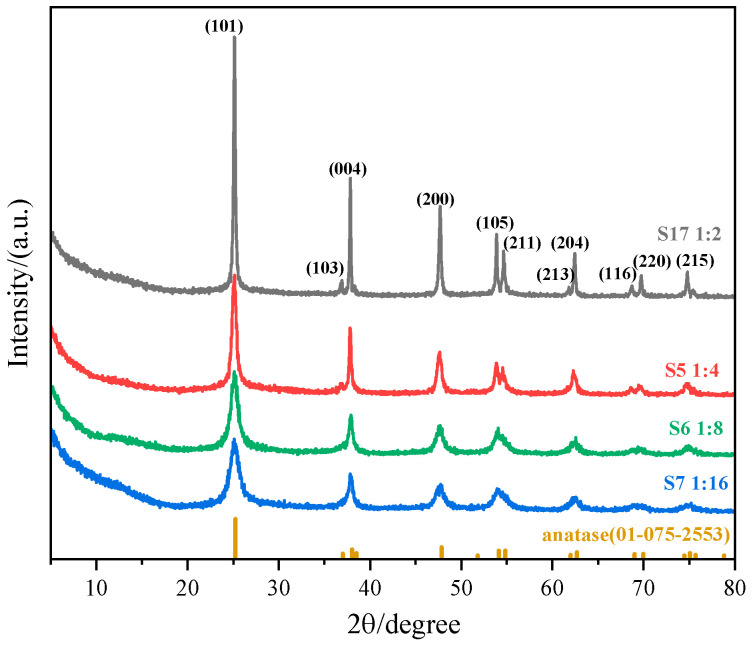
XRD pattern of sample nanoparticles synthesized under different molar ratios of (NH_4_)_2_TiF_6_ to ammonia solution (sample numbers S17, S5, S6, S7). Constant parameters maintained: (NH_4_)_2_TiF_6_ concentration = 0.01 mol·L^−1^, reaction temperature = 90 °C, reaction time = 1 h.

A fixed set of parameters ((NH_4_)_2_TiF_6_ concentration: 0.01 mol·L^−1^, reaction temperature: 90 °C, duration: 1 h) was employed for experiments S17, S5, S6, and S7, while systematically altering the molar ratio of (NH_4_)_2_TiF_6_ to ammonia. This controlled variation enables the direct assessment of how reactant proportions influence the morphology and size distribution of TiO_2_ nanoparticles (Figure 3), revealing their critical role in regulating crystallization. Figure 4 shows the XRD patterns of S17, S5, S6, and S7.

At lower molar ratios (1:2), the insufficient OH^−^ concentration in the solution retards the hydrolysis of [TiF_6_]^2−^, resulting in diminished nucleation rates and dominant crystal growth. Under such conditions, limited nuclei undergo growth via Ostwald ripening or oriented attachment mechanisms, resulting in the formation of larger particles. With increasing molar ratios, excess ammonia creates a highly alkaline environment that accelerates the hydrolysis of [TiF_6_]^2−^ into TiO_2_, where the nucleation rate exceeds the growth rate, thereby progressively reducing nanoparticle size (S7: ~25 nm).

The transmission electron microscopy (TEM) analysis of sample S17 revealed the presence of particles exhibiting a typical hierarchical assembly morphology (circled in the TEM image of sample S17 in Figure 3). These particles consist of several spindle-shaped units growing radially along specific crystallographic directions, forming a multi-layered, flower-like morphology. High-resolution TEM (HRTEM) confirmed that the particles are assembled from individual spindle-shaped units rather than simple stacking (Figure S5b,c). Selected area electron diffraction (SAED) revealed multiple sets of symmetrical diffraction spots (Figure S5d), possibly indicating the presence of twin structures within these multi-layered, flower-like assemblies. However, the particles themselves exhibit low symmetry, suggesting the coexistence of twin structures with other defect configurations, leading to imperfect symmetry. This aspect will not be further discussed here.

#### 3.1.2. Impact of Reaction Time

**Figure 5:**
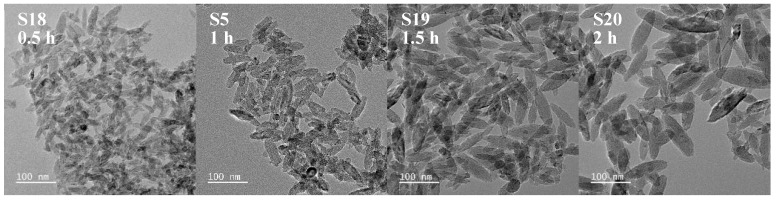
TEM images of spindle-shaped TiO_2_ nanoparticles synthesized at different reaction times (sample numbers S18, S5, S19, S20). The constant parameters maintained were: molar ratio of (NH_4_)_2_TiF_6_ to ammonia solution = 1:4, reaction temperature = 90 °C, and (NH_4_)_2_TiF_6_ concentration = 0.01 mol·L^−1^. Scale bar: 100 nm.

**Figure 6:**
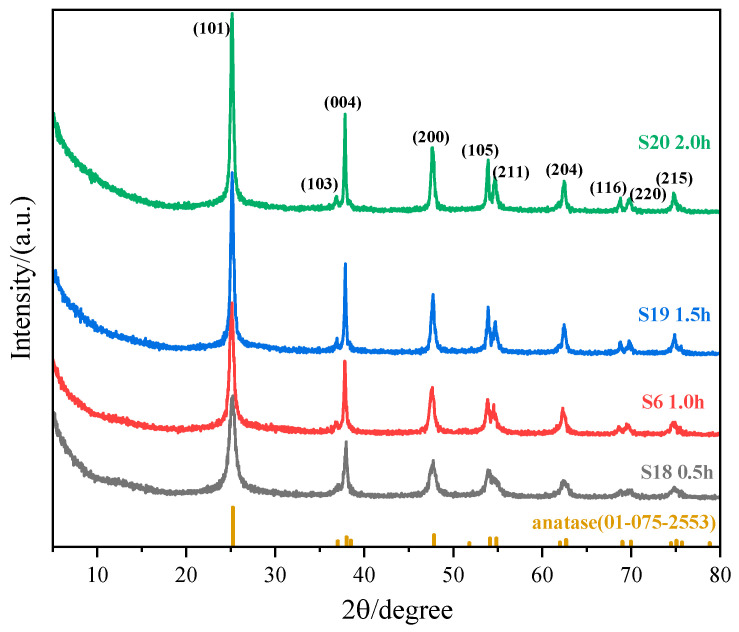
XRD pattern of sample nanoparticles synthesized at different reaction times (sample numbers S18, S5, S19, S20). The constant parameters maintained were: molar ratio of (NH_4_)_2_TiF_6_ to ammonia solution = 1:4, reaction temperature = 90 °C, and (NH4)2TiF6 concentration = 0.01 mol·L^−1^.

To isolate temporal effects, experiments S18, S5, S19, and S20 maintained consistent conditions (molar ratio: 1:4, temperature: 90 °C, (NH_4_)_2_TiF_6_ concentration: 0.01 mol·L^−1^) but differed in reaction durations. Through this approach, we examined the evolution of nanoparticle characteristics over time (Figure 5 and Figure 6) and analyzed how crystallization kinetics respond to extended processing periods.

All TiO_2_ nanoparticles exhibited spindle-shaped morphologies. Prolonging the reaction duration from 0.5 h to 2 h resulted in a progressive increase in particle size. Extended reaction durations correlated with slower titration rates, thereby maintaining a low precursor concentration environment over prolonged periods. Under these conditions, crystal growth rates exceeded nucleation rates (G > J). At the same time, Ostwald ripening became operative, as smaller particles dissolved and larger ones grew, leading to increased particle sizes with narrower size distributions. Conversely, shorter reaction times (e.g., 0.5 h) with rapid titration triggered burst nucleation, resulting in smaller particles with broader size distributions (Sample S18: 20–75 nm range).

#### 3.1.3. Effect of Reaction Temperature

**Figure 7:**
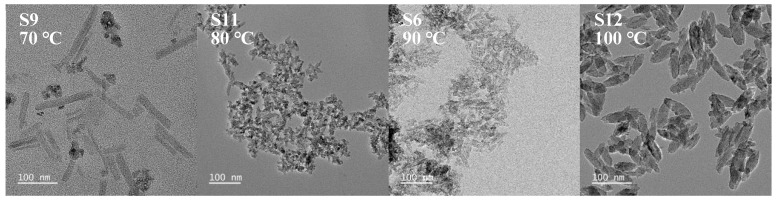
TEM images of spindle-shaped TiO_2_ nanoparticles synthesized at different reaction temperatures (sample numbers S9, S11, S6, S12). Constant parameters: (NH_4_)_2_TiF_6_ concentration = 0.01 mol·L^−1^, molar ratio of (NH_4_)_2_TiF_6_ to ammonia solution = 1:8, reaction time = 1 h. Note the rod-shaped morphology present in sample S9. Scale bar: 100 nm.

**Figure 8:**
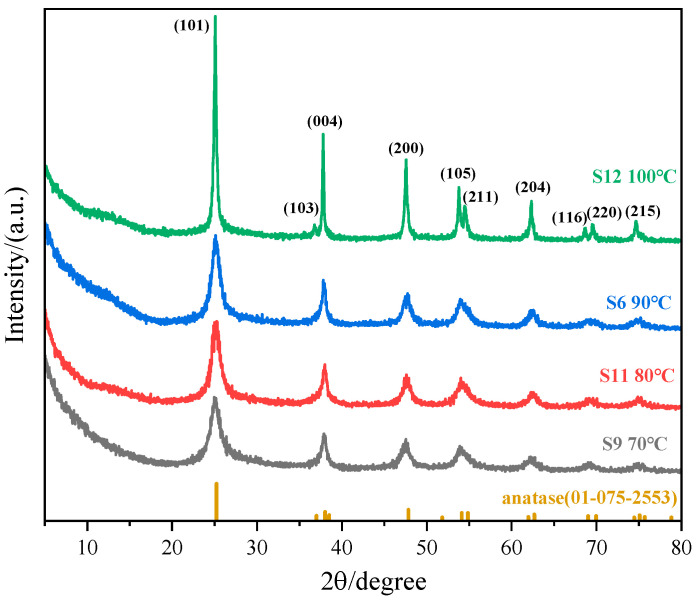
XRD pattern of sample nanoparticles synthesized at different reaction temperatures (sample numbers S9, S11, S6, S12). Constant parameters: (NH_4_)_2_TiF_6_ concentration = 0.01 mol·L^−1^, molar ratio of (NH_4_)_2_TiF_6_ to ammonia solution = 1:8, reaction time = 1 h.

Thermal influences on crystallization were probed in experiments S9, S11, S6, and S12. Holding the precursor concentration (0.01 mol·L^−1^), molar ratio (1:8), and duration (1 h) constant while adjusting the reaction temperature permitted a comparative evaluation. Figure 7 illustrates the morphology and size of TiO_2_ nanoparticles prepared at various temperatures, while Figure 8 presents their XRD pattern variations, enabling an analysis of how reaction temperature affects crystallization behavior.

With increasing reaction temperature, the kinetic energy barrier decreases, enabling accelerated hydrolysis and polycondensation reactions. Both nucleation and growth rates are enhanced at elevated temperatures. At lower temperatures (70 °C and 80 °C), nucleation dominates, yielding nanoparticles with smaller sizes (≤10 nm). Conversely, under higher-temperature conditions, growth-dominated mechanisms prevail. Thermal energy promotes crystallographic facet-selective growth, driving particle coarsening that progresses to sizes exceeding 100 nm.

At 70 °C, co-existing nanorod and spindle-shaped morphologies were observed. The nanorod morphology arises from preferential F^−^ adsorption (derived from the decomposition of (NH_4_)_2_TiF_6_) onto the high-energy {001} facets of anatase TiO_2_, which inhibits growth along this orientation while enabling rapid elongation along the low-energy {101} facets. At reduced temperatures (70 °C), F^−^ adsorption exhibits enhanced stability (ΔH > 0 for Equation (4), significantly lowering the surface energy of {001} facets and promoting anisotropic growth. The moderate concentration of TiO_2_ precursors generated via [TiF_6_]^2−^ hydrolysis at 70 °C creates localized zones that meet the nanorod growth criteria (F^−^-adsorption dominance), while other regions with insufficient F^−^ concentrations yield spindle-shaped particles. Additionally, fluctuations in local ammonia concentrations during titration may induce microdomain pH gradients, allowing for the selective crystallographic growth of nanorods in specific zones. At the same time, residual precursors form smaller, spindle-shaped particles, ultimately resulting in mixed morphological characteristics.

#### 3.1.4. Effect of (NH_4_)_2_TiF_6_ Concentration

**Figure 9:**
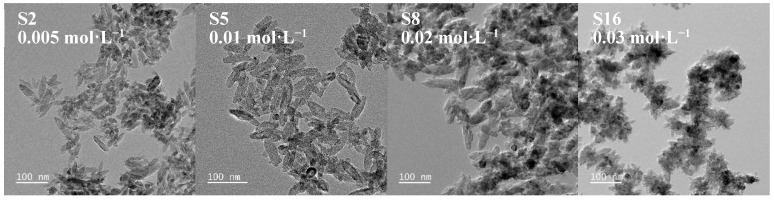
TEM images of spindle-shaped TiO_2_ nanoparticles synthesized at different (NH_4_)_2_TiF_6_ concentrations (sample numbers S2, S5, S8, S16). Constant parameters maintained: molar ratio of (NH_4_)_2_TiF_6_ to ammonia solution = 1:4, reaction temperature = 90 °C, reaction time = 1 h. Scale bar: 100 nm.

**Figure 10:**
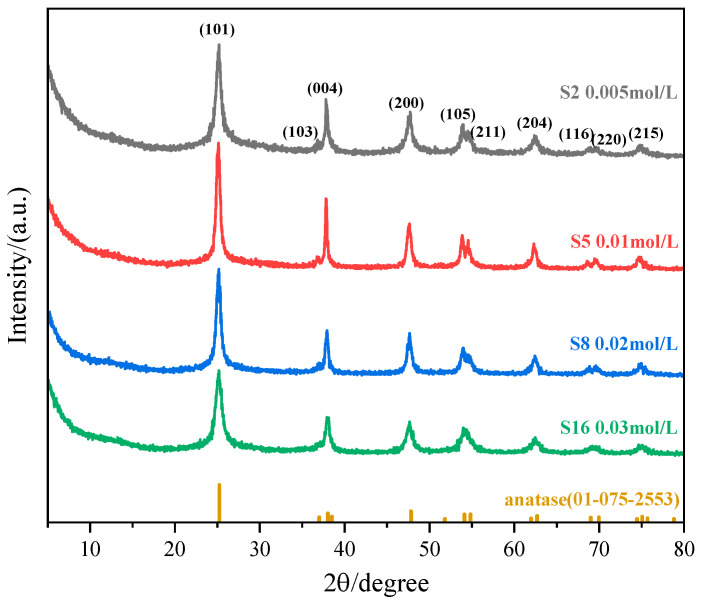
XRD pattern of sample nanoparticles synthesized at different (NH_4_)_2_TiF_6_ concentrations (sample numbers S2, S5, S8, S16). Constant parameters maintained: molar ratio of (NH_4_)_2_TiF_6_ to ammonia solution = 1:4, reaction temperature = 90 °C, reaction time = 1 h.

The impact of (NH_4_)_2_TiF_6_ concentration was investigated using samples S2, S5, S8, and S16 synthesized with identical reaction settings (molar ratio: 1:4, temperature: 90 °C, duration: 1 h). By comparing the morphological characteristics at different concentrations and XRD patterns (Figure 9 and Figure 10), we analyzed the regulatory effect of concentration on the crystallization behavior of nanoparticles.

The experimental results show that as the concentration of ammonium fluorotitanate increases gradually from 0.005 mol·L^−1^ to 0.03 mol·L^−1^, the obtained TiO_2_ particles all exhibit a spindle-like morphology; however, their particle size displays a non-monotonic trend, first increasing and then decreasing. At a lower concentration of ammonium fluorotitanate (0.005 mol·L^−1^), the concentration of [TiF_6_]^2−^ ions in the solution is low. Although micro-region burst nucleation occurs after the addition of ammonia water, the total number of crystal nuclei is significantly lower than that in high-concentration systems. Due to the limited initial number of nuclei and insufficient reaction time (1 h) for Ostwald ripening to proceed fully, particle growth primarily occurs through adsorption, resulting in smaller particle sizes. As the concentration increases (0.01 mol·L^−1^), the number of nuclei increases, and ripening becomes more complete within the same time, leading to relatively larger particle sizes. When the concentration of ammonium fluorotitanate rises to 0.02 mol·L^−1^, the concentration of TiO_2_ precursors generated by the hydrolysis of [TiF_6_]^2−^ increases but does not reach the critical supersaturation point. At this stage, nucleation and growth rates achieve a certain dynamic balance, allowing particles to grow continuously in a uniform environment, forming larger and more uniform crystals. However, when the concentration further increases (0.03 mol·L^−1^), the excessively high concentration of [TiF_6_]^2−^ ions in the solution leads to a sharp rise in local supersaturation, triggering burst nucleation and generating a large number of tiny crystal nuclei. Here, the nucleation rate far exceeds the growth rate, ultimately resulting in spindle-like particles with reduced sizes and broader size distributions.

### 3.2. Catalytic Activity

**Figure 11:**
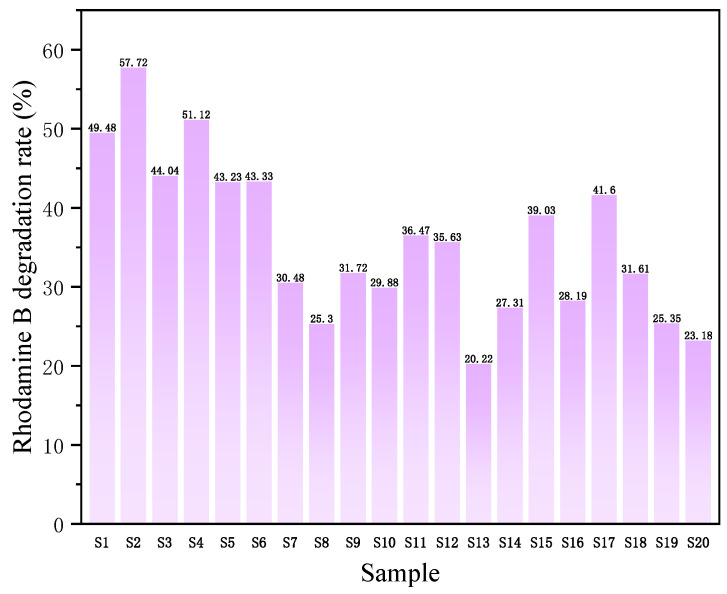
Bar chart of photocatalytic degradation efficiency.

The original absorbance data and calculation process are shown in Supplementary Information Table S5. The photocatalytic degradation efficiency data are summarized in Figure 11.

Table 1 presents the photocatalytic degradation efficiencies sorted in descending order alongside the finalized experimental matrix. As detailed in Section 2.4, coded values ranging from 0 to 4 were assigned to each input factor level. The response variables were binary, with values assigned as 0 or 1 based on photocatalytic degradation efficiency thresholds (Figure S1). To analytically interrogate this experimental matrix and establish input-output relationships, Equation (S1) was employed to calculate correlation coefficients. The computational results are summarized in Table 1. For each factor level, coefficients were determined to quantify its influence on output factors, where higher coefficient magnitudes indicate greater contributory impacts of the corresponding factor levels.

The contributions of each input factor level to output factors OF1 and OF2 were evaluated through comparative analysis of the coefficient values summarized in Table 2. Several key trends emerged from these results, with particular focus given to the input factor (IF) exhibiting the highest coefficient magnitude. The analysis of input factor (IF) results underscores the pivotal role of catalyst particle size. For output factor OF1 (top 50% degradation efficiency), Level 3 of IF exhibited the highest coefficient (0.67), followed by Level 1 (0.57) and Level 2 (0.50), all of which significantly surpassed Levels 4 (0.25) and 5 (0.00). This indicates that particle sizes within the 25–100 nm range markedly enhance photocatalytic performance. For OF2 (top 25% degradation efficiency), Level 3 again dominated with a coefficient of 0.5, suggesting that the optimal particle size for superior catalytic activity lies within the 50–75 nm range.

## 4. Conclusions

This study systematically investigates the effects of synthesis parameters on the particle size of nano-TiO_2_ photocatalysts. Subsequently, it correlates particle size with photocatalytic performance, thereby emphasizing the critical role of synthetic pathways. The study aimed to identify the experimental conditions that enable optimal photocatalytic performance in TiO_2_ nanoparticles synthesized via ammonium fluorotitanate-ammonia precipitation. To maximize information yield while minimizing the number of experimental iterations, an experimental design methodology was implemented. Multiple combinations of preparation parameters were systematically explored during the synthetic process. The synthesized powder was analyzed by X-ray diffraction and further confirmed by HRTEM images: the sample contains some amorphous material, with diffraction peaks entirely corresponding to the anatase phase, and no other crystalline impurity peaks were observed. Transmission electron microscopy observations further confirmed the structural integrity and morphological uniformity of the product.

The synthesized particles predominantly exhibited spindle-shaped morphologies, with nanorod structures emerging under specific conditions. Low molar ratios (1:2) or prolonged reaction durations (2 h) promoted particle growth through nucleation suppression and Ostwald ripening, resulting in larger particles (>100 nm). Conversely, high molar ratios (1:16), short reaction times (0.5 h), or low temperatures (70 °C) accelerated nucleation while limiting growth, producing smaller particles (<25 nm). At low (NH_4_)_2_TiF_6_ concentrations (0.005 mol·L^−1^), sparse nucleation resulted in small particles, whereas intermediate concentrations (0.01–0.02 mol·L^−1^) balanced nucleation and growth, generating uniform, large particles. Elevated temperatures (90–100 °C) intensified particle coarsening. Nanorod formation required combined low-temperature conditions (70 °C) and facet-selective F^−^ adsorption to stabilize specific crystallographic orientations. Strategic parameter combinations—such as high molar ratio, low temperature, and short duration for ultrasmall particles, or low molar ratio, high temperature, and extended time for larger particles—enabled the precise design of TiO_2_ nanocrystals with sizes spanning 10–120 nm and tailored morphologies (spindles and rods).

Data acquired from TEM analysis facilitated the construction of the experimental matrix by identifying output factors, where input factors were defined based on particle size distribution characteristics. Response coefficients were calculated to evaluate the positive or negative impacts of particle size on optimal photocatalytic performance. The derived correlation coefficients revealed that particles within the 25–100 nm size range exhibited significantly enhanced photocatalytic activity compared to those below 25 nm or exceeding 100 nm, with the 50–75 nm sub-range demonstrating optimal catalytic efficiency.

## Figures and Tables

**Table 1:** Experimental matrix containing the coded values defined for input factors (IF) and output factors (OF).

Sample	IF	Degradation Rate Ranking	OF1	OF2
S2	3	1	1	1
S4	1	2	1	1
S1	4	3	1	1
S3	1	4	1	1
S6	2	5	1	1
S5	1	6	1	0
S17	4	7	1	0
S15	0	8	1	0
S11	1	9	1	0
S12	3	10	1	0
S9	0	11	0	0
S18	1	12	0	0
S7	2	13	0	0
S10	1	14	0	0
S16	0	15	0	0
S14	0	16	0	0
S19	3	17	0	0
S8	4	18	0	0
S20	4	19	0	0
S13	1	20	0	0

**Table 2:** Computed coefficients. Each coefficient indicates the influence of the input factor (IF) level on the output factors (OF1 and OF2). The highest values are in bold.

OF1 Top 50%	0	1	2	3	4
Factor/Level					
IF	0.25	0.57	0.50	**0.67**	0.00
OF2 Top 25%					
Factor/Level	0	1	2	3	4
IF	0.00	0.29	**0.50**	0.33	0.00

## Data Availability

The datasets generated and/or analyzed during the current study are not publicly available due to significant technical complexities and the specific expertise required for their preparation and accessibility beyond the scope of the current analysis. However, the data supporting the findings of this study are available from the corresponding author upon reasonable request. Requests will be evaluated for scientific merit and compliance with ethical agreements.

## Supplementary Materials

The following supporting information can be downloaded at: https://www.mdpi.com/article/10.3390/nano15120930/s1, Table S1. Sample codes and experimental parameters.; Figure S1. Input-output factors (IF and OF) in the experimental design methodology. A single input factor (IF) with four levels is assigned coded values (0, 1, 2, or 3). The output response (OF) exhibits binary behavior (0 or 1).; Table S2. Schematic representation of the experimental matrix with coded values Xk and Yj/k for input-output factors (IF = input factor, OF = output factor). Subscripts j and k denote the number of output factors (j = 1 or 2) and synthesis sequence (1 < k < n), respectively. Coded values are defined as: Xk = 0, 1, 2, or 3; Yj/k = 0 or 1; Table S3. Example of a matrix of computed coefficients Cj(L) obtained for an output factor j. Each coefficient indicates the influence of the level L (0 ≤ L ≤ 4) of the input factor on the output factor j.; Figure S2. UV-Vis absorption spectrum of Rhodamine B with λmax at 554 nm. (Concentration: 5 mg/L, Scan range: 200–700 nm, Solvent: H_2_O); Table S4. Absorbance changes and degradation rates of Rhodamine B solution photocatalyzed by P25 (Initial concentration: 20 mg/L, pre-reaction solution diluted by 4×, A_0_ = 2.8844); Table S5. Changes in absorbance and degradation rate of rhodamine B solution under photocatalytic conditions for one hour for all samples (experiments were conducted on different days, with the rhodamine B solution diluted fourfold before each experiment and the initial absorbance A0 measured).; Figure S3. Transmission electron microscopy (TEM) images of titanium dioxide samples, showcasing the spindle-shaped morphology of nanoparticles in samples S1–S20, as well as the rod-like structures present in S9 nanoparticles. Scale bar: 100 nm.; Figure S4. TEM and HRTEM images of sample S2. Figure (a) shows the TEM image with a scale bar of 100 nm, while figures (b), (c), and (d) display HRTEM images with clearly visible lattice fringes. The scale bars are 10 nm for figure (b) and 5 nm for figures (c) and (d).; Figure S5. TEM and HRTEM images of sample S17. Figure (a) shows a TEM image with a scale bar of 50 nm, while figures (b) and (c) display HRTEM images with clearly visible lattice fringes, each with a scale bar of 10 nm. Figure (d) presents the corresponding selected area electron diffraction (SAED) pattern of Figure (c), where multiple sets of symmetrical diffraction spots can be observed. Figure S6. XPS fine spectra of F1s (a) and N1s (b) for sample S2. The binding energies at two positions in the figure represent the bonding energy of F-metal bonds (684.3 eV) and N-H bonds (400.1 eV), respectively.
