# Supplementary material for: Synthesized Nano-Titanium Dioxide (Nano-TiO2) via Ammonium Fluorotitanate ((NH4)2TiF6) Precipitation with Ammonia Solution
Source: Nanomaterials (Basel). 2025 Jun 15;15(12):930. doi: 10.3390/nano15120930 (PMC12195784; doi:10.3390/nano15120930)
Supplement: Supplementary file 1 [file nanomaterials-15-00930-s001.zip › nanomaterials-3680668-supplementary.pdf]

Part 1 Supplementary Experimental Parameters and Methods

1.1 Experimental Parameters

Table S1 shows the synthesis parameters and sample numbers:

Table S1. Sample codes and experimental parameters

Sample	$(NH_4)_2TiF_6$ concentration ($mol{\cdot}L^{-1}$)	$NH_4{\cdot}H_2O$ concentration ($mol{\cdot}L^{-1}$)	$(NH_4)_2TiF_6$:$NH_4{\cdot}H_2O$	temperature (°C)	time (h)
S1	0.005	0.01	1:2	90	1
S2	0.005	0.02	1:4	90	1
S3	0.005	0.04	1:8	90	1
S4	0.005	0.08	1:16	90	1
S5	0.01	0.04	1:4	90	1
S6	0.01	0.08	1:8	90	1
S7	0.01	0.16	1:16	90	1
S8	0.02	0.08	1:4	90	1
S9	0.01	0.08	1:8	70	1
S10	0.01	0.08	1:8	70	2
S11	0.01	0.08	1:8	80	1
S12	0.01	0.08	1:8	100	1
S13	0.02	0.16	1:8	90	1
S14	0.02	0.32	1:16	90	1
S15	0.03	0.48	1:16	90	1
S16	0.03	0.12	1:4	90	1
S17	0.01	0.02	1:2	90	1
S18	0.01	0.04	1:4	90	0.5
S19	0.01	0.04	1:4	90	1.5
S20	0.01	0.04	1:4	90	2

1.2 An experimental design inspired by the Taguchi method

In this study, the controlled input factor (IF) was defined as particle size, while the measured output factor (OF) corresponded to photocatalytic performance, as schematically illustrated in Figure S1. The input parameter was divided into five levels (unit: nm): 0–25, 25–50, 50–75, 75–100, and >100, with coded values from 0 to 4 assigned to each level. Experimental results from photocatalytic evaluations enabled the definition of output responses, specifically the photocatalytic performance ranking of each tested sample within the dataset. Since the response variable was binary, two coded values (0 or 1) were adopted. A value of 1 indicated that the photocatalytic performance ranked within the top x% of all samples, whereas a value of 0 denoted

lower rankings. Two output factors were selected: OF1 and OF2, representing the top 50% and top 25% performance rankings, respectively. Coded values associated with input and output factors were utilized to construct the experimental matrix, as illustrated in Table S2.

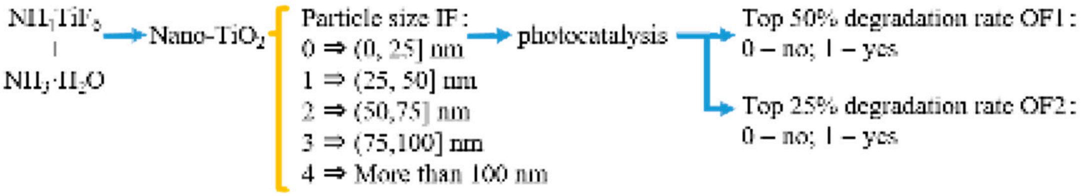

Figure S1. Input-output factors (IF and OF) in the experimental design methodology. A single input factor (IF) with four levels is assigned coded values (0, 1, 2, or 3). The output response (OF) exhibits binary behavior (0 or 1).

To evaluate the influence of each input factor level on the output factor (OF), a binary variable $Z_k(L)$ was introduced, where Zk(L) = 1 when Xk = L, and Zk(L) = 0 otherwise. Equation (5) was applied to calculate the coefficient $C^j(L)$ for each level of the input factors, thereby establishing correlations between the output and input factors.

$$C^j(L) = \frac{1}{N}\sum_k Z_k\,(L) \times Y_{j/k} \qquad \text{(S1)}$$

Table S2. Schematic representation of the experimental matrix with coded values X_k and $Y_{j/k}$ for input-output factors (IF = input factor, OF = output factor). Subscripts j and k denote the number of output factors (j = 1 or 2) and synthesis sequence (1 < k < n), respectively. Coded values are defined as: X_k = 0, 1, 2, or 3; $Y_{j/k}$ = 0 or 1.

Factor/Synthesis	IF	OF1	OF2
S_1	X_1	$Y_{1/1}$	$Y_{2/1}$
S_2	X_2	$Y_{1/2}$	$Y_{2/2}$
.	.	.	.
S_k	X_k	$Y_{1/k}$	$Y_{2/k}$
.	.	.	.
S_n	X_n	$Y_{1/n}$	$Y_{2/n}$

In equation (S1), the symbols j, k, and L correspond to the output factor number (j = 1 or 2), synthesis index (1 ≤ k ≤ 20), and factor level index (0 ≤ L ≤ 4), respectively. The parameter N represents the occurrence frequency of L in the $|X_{i/k}|$ column. For the output factors under study, we derived a coefficient matrix similar to the one shown in Table S3. The larger the coefficient of an input factor for a given output factor, the greater the contribution of that input parameter to the output factor.

Table S3. Example of a matrix of computed coefficients $C^j(L)$ obtained for an output factor j.

Each coefficient indicates the influence of the level L (0≤L≤4) of the input factor on the output factor j.

Factor/Level	0	1	2	3	4
IF	$C^j(0)$	$C^j(1)$	$C^j(2)$	$C^j(3)$	$C^j(4)$

Part 2 Supplementary Absorbance Data

Before the photocatalytic experiment began, the maximum absorption wavelength of Rhodamine B was measured at 554 nm (Figure S2). Therefore, the maximum absorbance of Rhodamine B was measured at 554 nm before and after the photocatalytic experiment.

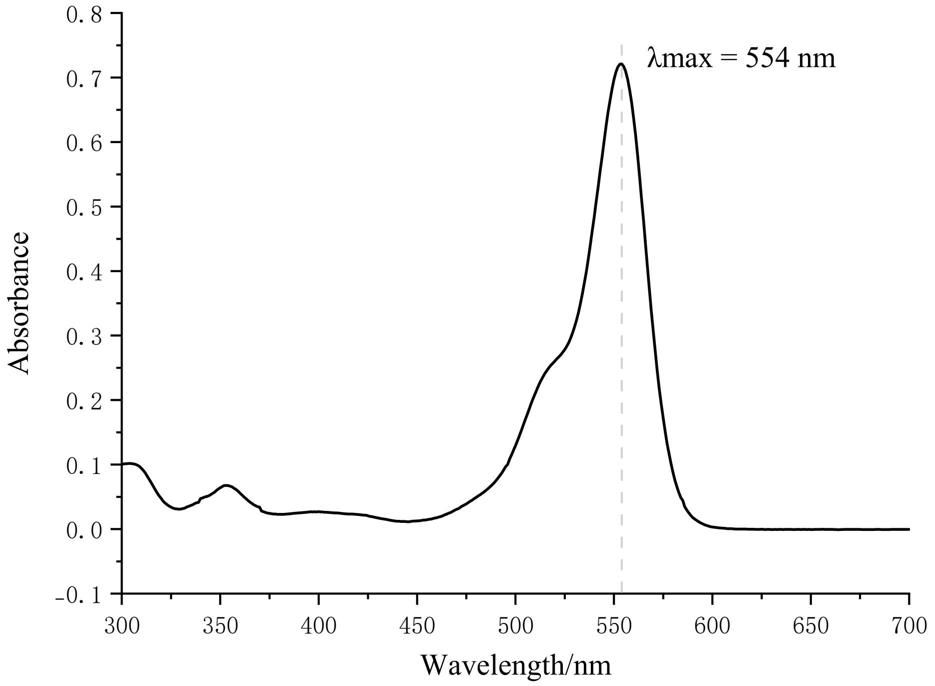

Figure S2. UV-Vis absorption spectrum of Rhodamine B with λmax at 554 nm. (Concentration: 5 mg/L, Scan range: 200–700 nm, Solvent: H_2O)

Before the formal experiment, the photocatalytic performance of commercially available P25 nano-titanium dioxide (referred to as P25) was verified. Specifically, 25 mL of Rhodamine B solution and 0.01 g of P25 catalyst were ultrasonically mixed in a dark environment for ten minutes, followed by photocatalytic degradation under the irradiation of a 365 nm wavelength, 30 W low-pressure mercury lamp for one hour. Every half hour, a portion of the solution was removed, and after high-speed centrifugation to separate P25, the supernatant was retested for absorbance. Table S4

shows the changes in absorbance and the corresponding degradation rates. It is worth mentioning that in scientific experiments, absorbance should preferably be controlled between 0.2 and 0.8, where the data has good linearity and minimal error, making it suitable for quantitative analysis. The initial concentration of Rhodamine B used in the experiment was 20 mg/L, which exceeded this absorbance range. After multiple adjustments, the final testing protocol involved diluting the initial absorbance by a factor of four and the post-reaction solution absorbance by a factor of four or higher before testing.

Table S4. Absorbance changes and degradation rates of Rhodamine B solution photocatalyzed by P25 (Initial concentration: 20 mg/L, pre-reaction solution diluted by 4×, A_0=2.8844)

Reaction time/h	0.5	1	1.5	2
Absorbance	0.519	0.4116	0.3132	0.1003
Dilution factor	4	4	4	3
Post-reaction absorbance (A)	2.076	1.6464	1.2528	0.3009
Degradation rate/%	28.027	42.921	56.566	86.091

Note: 1. The absorbance measured after 4-fold dilution of the pre-reaction solution was 0.7211, so the original $A_0 = 0.7211 \times 4 = 2.8844$.

2. The "Absorbance" column after the reaction shows the measured values after dilution, while "Post-reaction absorbance" represents the calculated values before dilution (= Absorbance × Dilution factor).

3. Degradation rate calculation formula: $\eta = [(A_0 - A)/A_0] \times 100\%$, where A is the absorbance before dilution after the reaction (i.e., the "Post-reaction absorbance" column).

During the formal experiment, the reaction time was uniformly set at one hour. Table S5 shows the absorbance and dilution factors of each sample before and after the reaction. In the table, A0 = dilution factor of the solution before reaction * absorbance of the diluted solution; A = dilution factor of the solution after one hour of reaction * absorbance of the diluted solution; Absorbance= $[(A_0 - A)/A_0] \times 100\%$

Table S5. Changes in absorbance and degradation rate of rhodamine B solution under photocatalytic conditions for one hour for all samples (experiments were conducted on different days, with the rhodamine B solution diluted fourfold before each experiment and the initial absorbance A0 measured).

Sample	Experiment date	Before the reaction			After 1 hour of reaction			
		Dilution factor	Measured absorbance	Absorbance A0	Dilution factor	Measured absorbance	Absorbance A	Degradation rate/%
A1	day 1	4	0.7209	2.8836	4	0.3567	1.4268	49.480
A2	day 1	4	0.7209	2.8836	4	0.4161	1.6644	57.720
A3	day 1	4	0.7209	2.8836	4	0.3175	1.2699	44.040
A4	day 1	4	0.7209	2.8836	4	0.3685	1.4741	51.120
A5	day 1	4	0.7209	2.8836	4	0.3116	1.2465	43.227
A6	day 2	4	0.7225	2.8900	4	0.3130	1.2521	43.326
A7	day 3	4	0.7288	2.9152	4	0.2222	0.8886	30.483
A8	day 4	4	0.7240	2.8960	4	0.1832	0.7327	25.301
A9	day 2	4	0.7225	2.8900	4	0.2291	0.9166	31.716
A10	day 5	4	0.7196	2.8784	4	0.2151	0.8602	29.885
A11	day 3	4	0.7288	2.9152	4	0.2658	1.0631	36.469
A12	day 2	4	0.7225	2.8900	4	0.2574	1.0297	35.630
A13	day 5	4	0.7196	2.8784	4	0.1455	0.5821	20.224
A14	day 3	4	0.7288	2.9152	4	0.1990	0.7960	27.306
A15	day 3	4	0.7288	2.9152	4	0.2845	1.1379	39.035
A16	day 4	4	0.7240	2.8960	4	0.2041	0.8165	28.193
A17	day 3	4	0.7288	2.9152	4	0.3032	1.2127	41.600
A18	day 6	4	0.7115	2.8460	4	0.2249	0.8995	31.607
A19	day 6	4	0.7115	2.8460	4	0.1803	0.7214	25.348
A20	day 6	4	0.7115	2.8460	4	0.1650	0.6598	23.184

Part 3 Supplementary TEM Images and SAED Diffraction Patterns

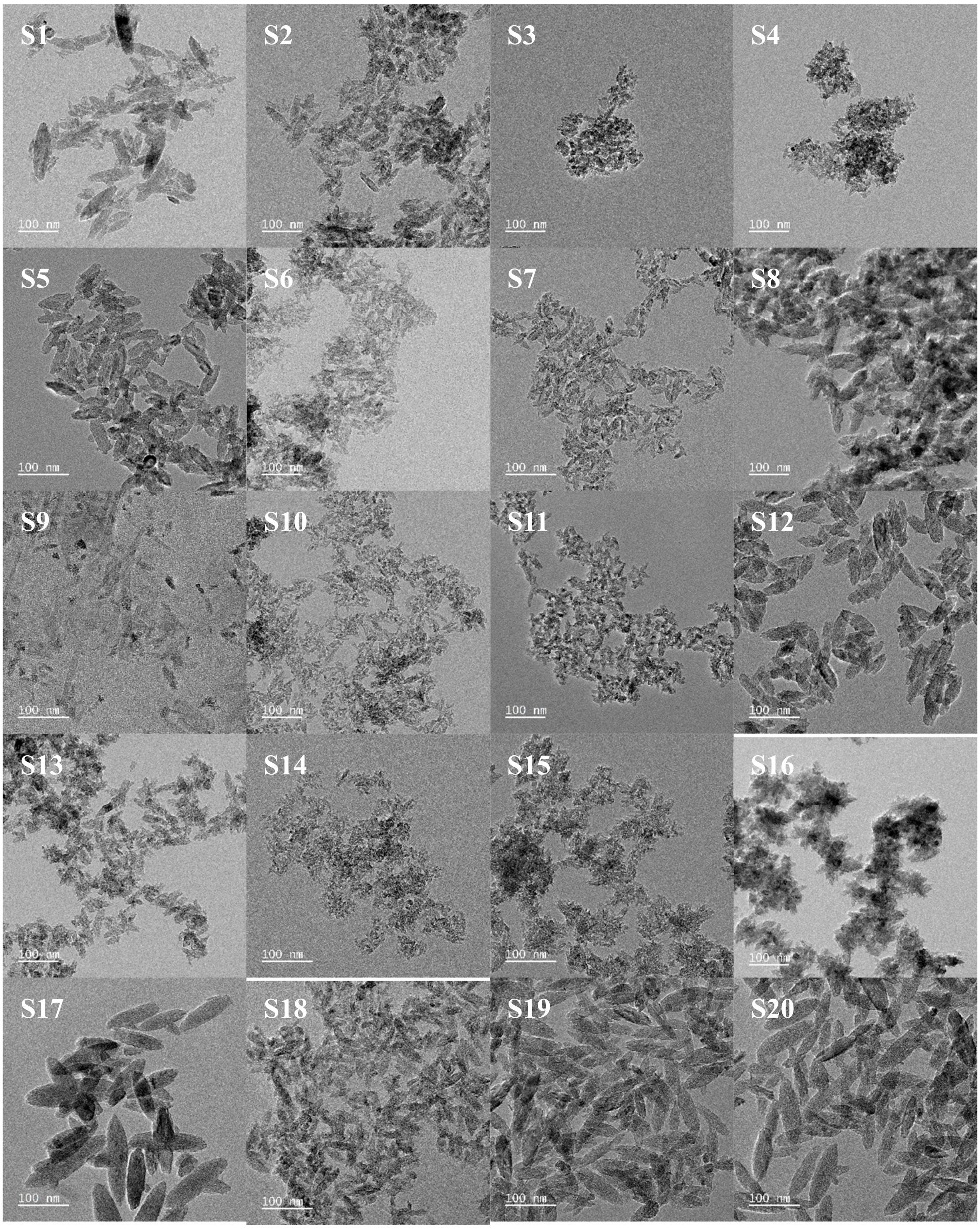

Figure S3. Transmission electron microscopy (TEM) images of titanium dioxide samples, showcasing the spindle-shaped morphology of nanoparticles in samples S1-S20, as well as the rod-like structures present in S9 nanoparticles. Scale bar: 100 nm.

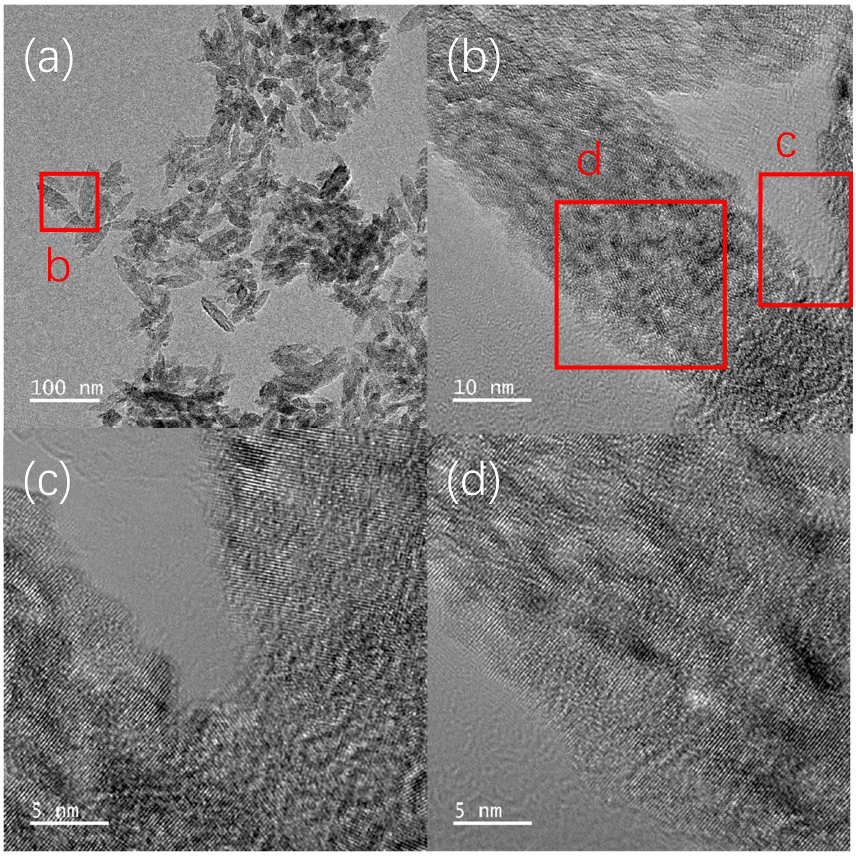

Figure S4. TEM and HRTEM images of sample S2. Figure (**a**) shows the TEM image with a scale bar of 100 nm, while figures (**b–d**) display HRTEM images with clearly visible lattice fringes. The scale bars are 10 nm for figure (**b**) and 5 nm for figure (**c**,**d**).

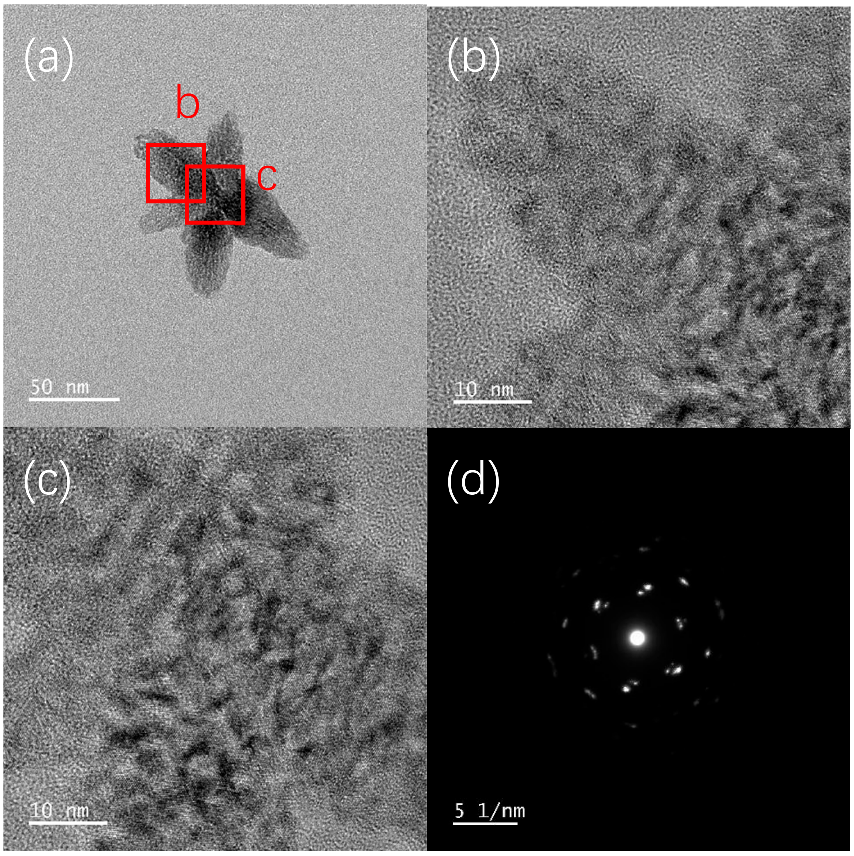

Figure S5. TEM and HRTEM images of sample S17. Figure (**a**) shows a TEM image with a scale bar of 50 nm, while figures (**b**,**c**) display HRTEM images with clearly visible lattice fringes, each with a scale bar of 10 nm. Figure (**d**) presents the corresponding selected area electron diffraction (SAED) pattern of Figure (**c**), where multiple sets of symmetrical diffraction spots can be observed.

Part 4 Supplementary X-ray Photoelectron Spectroscopy (XPS) Analysis

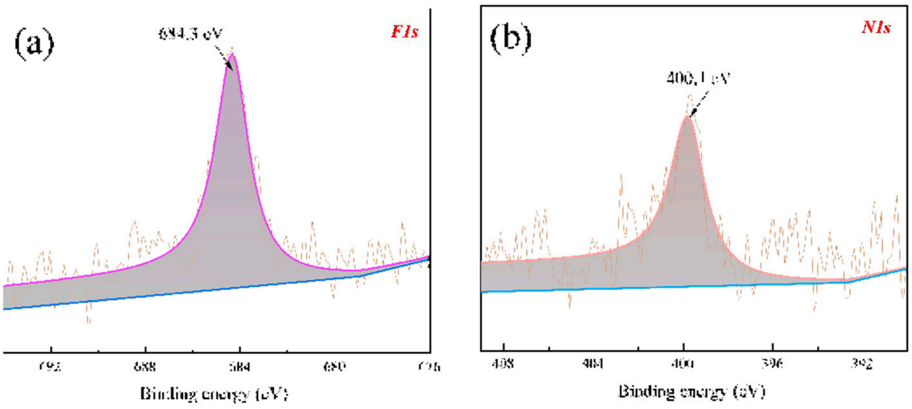

Figure S6. XPS fine spectra of F1s (**a**) and N1s (**b**) for sample S2. The binding energies at two positions in the figure represent the bonding energy of F-metal bonds (684.3 eV) and N-H bonds (400.1 eV), respectively.
